# Neutrophil exhaustion and impaired functionality in psoriatic arthritis patients

**DOI:** 10.3389/fimmu.2024.1448560

**Published:** 2024-09-06

**Authors:** Luca Modestino, Manuela Tumminelli, Ilaria Mormile, Leonardo Cristinziano, Annagioia Ventrici, Marialuisa Trocchia, Anne Lise Ferrara, Francesco Palestra, Stefania Loffredo, Gianni Marone, Francesca Wanda Rossi, Amato de Paulis, Maria Rosaria Galdiero

**Affiliations:** ^1^ Department of Internal Medicine and Clinical Immunology, University Hospital of Naples Federico II, Naples, Italy; ^2^ Department of Translational Medical Sciences (DiSMeT), University of Naples Federico II, Naples, Italy; ^3^ Center for Basic and Clinical Immunology Research (CISI), University of Naples Federico II, Naples, Italy; ^4^ Institute of Experimental Endocrinology and Oncology ‘G. Salvatore’, National Research Council (CNR), Naples, Italy

**Keywords:** neutrophils, neutrophil extracellular traps, psoriatic arthritis, inflammation, innate immunity

## Abstract

**Background:**

Neutrophils (polymorphonuclear leukocytes, PMNs) are the most abundant subtype of white blood cells and are among the main actors in the inflammatory response. Psoriatic arthritis (PsA) is a chronic inflammatory disease affecting both the axial and peripheral joints. Typically associated with psoriasis, PsA can also affect multiple systems and organs, including the nails and entheses. Despite the involvement of PMNs in PsA, their specific role in the disease remains poorly understood. This study aimed to characterize the biological functions of PMNs and neutrophil-related mediators in PsA patients.

**Materials and methods:**

31 PsA patients and 22 healthy controls (HCs) were prospectively recruited. PMNs were isolated from peripheral blood and subjected to *in vitro* stimulation with lipopolysaccharide (LPS), N-Formylmethionyl-leucyl-phenylalanine (fMLP), tumor necrosis factor (TNF), phorbol 12-myristate 13-acetate (PMA), or control medium. Highly purified peripheral blood PMNs (>99%) were evaluated for activation status, reactive oxygen species (ROS) production, phagocytic activity, granular enzyme and neutrophil extracellular traps (NETs) release. Serum levels of matrix metalloproteinase-9 (MMP-9), myeloperoxidase (MPO), TNF, interleukin 23 (IL-23), and interleukin 17 (IL-17) were measured by ELISA. Serum Citrullinated histone H3 (CitH3) was measured as a NET biomarker.

**Results:**

Activated PMNs from PsA patients displayed reduced activation, decreased ROS production, and impaired phagocytic activity upon stimulation with TNF, compared to HCs. PMNs from PsA patients also displayed reduced granular enzyme (MPO) and NET release. Serum analyses revealed elevated levels of MMP-9, MPO, TNF, IL-23, IL-17, and CitH3 in PsA patients compared to HCs. Serum CitH3 levels positively correlated with MPO and TNF concentrations, and IL-17 concentrations were positively correlated with IL-23 levels in PsA patients. These findings indicate that PMNs from PsA patients show reduced *in vitro* activation and function, and an increased presence of neutrophil-derived mediators (MMP-9, MPO, TNF, IL-23, IL-17, and CitH3) in their serum.

**Conclusions:**

Taken together, our findings suggest that PMNs from PsA patients exhibit an “exhausted” phenotype, highlighting their plasticity and multifaceted roles in PsA pathophysiology.

## Introduction

Psoriatic arthritis (PsA) is a heterogeneous, chronic, immune-mediated disease characterized by inflammation of the musculoskeletal system, including arthritis, enthesitis, spondylitis, and dactylitis. It commonly occurs in patients with psoriasis (PsO), with approximately 30% of PsO patients developing PsA (1). PsO typically precedes the onset of arthritis by an average of 10 years; however, in 15% of cases, arthritis and psoriasis occur simultaneously or arthritis precedes the skin manifestations (2). Moreover, around 15% of PsO patients have undiagnosed PsA (3). PsA can also be associated with uveitis, inflammatory bowel disease (IBD), cardiovascular disease, metabolic syndrome, obesity, diabetes mellitus, dyslipidemia, osteoporosis, and depression (4).

The pathogenesis of PsA is complex and multifaceted, consisting of an interplay between genetic predisposition, environmental triggers, and activation of the innate and adaptive immune response, culminating in inflammatory processes (5). Cytokines such as IL-17, IL-23, and TNF are major players in PsA, promoting the activation of endothelial cells, macrophages, fibroblasts, keratinocytes, dendritic cells, epithelial cells, chondrocytes, osteoclasts, and osteoblasts. Innate immune cells play a pivotal role in the pathogenesis of inflammatory arthritis, orchestrating the inflammatory cascade and its resolution at various levels (6, 7). The activation of immune cells leads to synovitis, enthesitis, erosions, and lesions in articular cartilage and skin (1).

Polymorphonuclear neutrophils (PMNs) contribute to the pathogenesis of PsA, although their role has not yet been fully described (8, 9). PMNs are present in typical psoriatic lesions, particularly in the epidermis, where they accumulate in Munro’s microabscesses (MMs) in the stratum corneum (10). In addition, recent studies have shown that psoriatic PMNs participate in both the initiation and maintenance phases of disease pathogenesis through the release of granular components, neutrophil extracellular traps (NETs), and the induction of oxidative stress (11). PMNs from patients with severe psoriasis display higher surface expression of CD66b and CD11b, and lower expression of CD62L compared to patients with moderate psoriasis and healthy controls (HCs)—an effect that is reversed by biological therapy (12).

In addition to the release of granular enzymes, phagocytosis, and the production of reactive oxygen species (ROS), PMNs release NETs to kill microorganisms (13). NETs are composed of nuclear molecules (DNA and histones) and granular enzymes, such as neutrophil elastase (NE), myeloperoxidase (MPO), pentraxin-3 (PTX3), and are released by activated PMNs in response to various immunological and non-immunological stimuli, including bacteria (14), fungi (15), and viruses (16, 17). NETs can also serve as a source of autoantigens, potentially contributing to the development of autoimmune diseases, including PsA (9, 18).

In this study, we aimed to evaluate PMNs and NETs in PsA patients. To this end, we conducted an *ex vivo* evaluation of the activation status, phagocytic activity, ROS production, and release of granular enzymes and NETs from highly purified peripheral blood PMNs from PsA patients compared to HCs. Additionally, we measured the circulating levels of soluble neutrophil-related mediators and NET-derived biomarkers in PsA patients and HCs.

## Materials and methods

### Patient recruitment

In this study, 31 PsA patients and 22 HCs, matched for sex and age, were recruited at the Division of Clinical Immunology and Internal Medicine of the University of Naples Federico II (Naples, Italy). The study was conducted in accordance with Good Clinical Practice (GCP) guidelines and adhered to the Declaration of Helsinki. Written informed consent was obtained from all participants. Patients were eligible for enrollment in the study if they were aged 18–70 years and had a diagnosis of PsA according to ClASsification criteria for Psoriatic ARthritis (CASPAR) criteria: evidence of psoriasis (or family history of psoriasis), possible psoriatic nail dystrophy, negative test for rheumatoid factor, dactylitis (current or previous history), and radiological evidence of juxta-articular new bone formation. The main exclusion criteria comprised other autoimmune diseases, infections, and malignancies. Peripheral blood samples were collected at the Center for Basic and Clinical Immunology Research (CISI) at the University of Naples Federico II and were immediately processed. Serum samples were obtained by centrifugation (+4°C, 400×g, 20 min) and stored at -80°C until use.

### Neutrophil purification and culture

The study procedure, involving the use of human blood cells, was approved by the Ethics Committee of the University of Naples Federico II (approval no. 194/2023). Leukocytes from peripheral blood samples of PsA patients and HCs were isolated from erythrocytes using HetaSep™ Solution (StemCell Technologies, Vancouver, Canada). PMNs were further purified by density gradient centrifugation using Lymphoprep™ (StemCell Technologies, Vancouver, Canada) at 400×g for 20 min at +22°C. To achieve >99% purity, PMNs were isolated from granulocytes through negative selection using the EasySep Neutrophil Enrichment Kit (StemCell Technologies, Vancouver, Canada) (19). Purity (>99% PMNs) was confirmed by flow cytometric analysis using the following antibodies: anti-CD3, anti-CD14, anti-CD15, anti-CD11b, anti-CD193 (Miltenyi Biotec, Bergisch Gladbach, Germany), anti-CD62L (L-selectin; BD Biosciences, San Jose, CA, USA), and anti-CD66b (BioLegend, San Diego, CA, USA). Samples were analyzed on the MACSQuant Analyzer 10 (Miltenyi Biotec, Bergisch Gladbach, Germany) and data were processed using FlowJo software (v.10). Dead cells, doublets, debris, and eosinophils were excluded from the analysis. Representative flow cytometric panels and the complete gating strategy used to verify PMN purity are illustrated in **Supplementary Figure 1**. Data are expressed as the percentage of positive cells (20).

### Flow cytometry

PMNs were maintained in RPMI supplemented with 10% of fetal bovine serum (FBS; Euroclone, Milan, Italy) and antibiotics at +37°C for 30 min, then were washed with phosphate-buffered saline (PBS). After washing, cells were stimulated with LPS (100 ng/mL), fMLP (1 µM), TNF (10 ng/mL), PMA (10 ng/mL), and/or control medium (RPMI 1640 medium with 5% FBS; Euroclone, Milan, Italy) as a negative control for 60 min at +37°C. Subsequently, 1.5 × 10^5^ cells were seeded in a 96-well plate (Thermo Fisher Scientific, Waltham, MA, USA) and incubated with Zombie Violet dye (BioLegend, San Diego, CA, USA) for 20 minutes at +4°C to evaluate cell viability. Following incubation, the cells were stained for 20 minutes at +4°C in PBS containing 1% FBS with the following antibodies: allophycocyanin (APC)-conjugated anti-CD66b (clone REA306, dilution 1:50; Miltenyi Biotec, Bergisch Gladbach, Germany), VioBlue-conjugated anti-CD193 (CCR3) (clone REA574, 1:10; Miltenyi Biotec, Bergisch Gladbach, Germany), peridinin chlorophyll protein (PerCP)-conjugated anti-CD11b (clone REA713, dilution 1:50; Miltenyi Biotec, Bergisch Gladbach, Germany), and fluorescein isothiocyanate (FITC)-conjugated anti-CD62L (clone 145/15, dilution 1:50; Miltenyi Biotec, Bergisch Gladbach, Germany). Cells were acquired using a MACS Quant Analyzer 10 (Miltenyi Biotec, Bergisch Gladbach, Germany) and analyzed with FlowJo software (v.10). Doublets and debris were excluded based on forward and side scatter properties, dead cells were excluded using the Zombie Violet Fixable Viability Kit (BioLegend, San Diego, CA, USA, and eosinophils were excluded based on the CCR3 exclusion gate. All experiments were performed in duplicate.

### Cytometric analytical strategy

**Figure 1:**
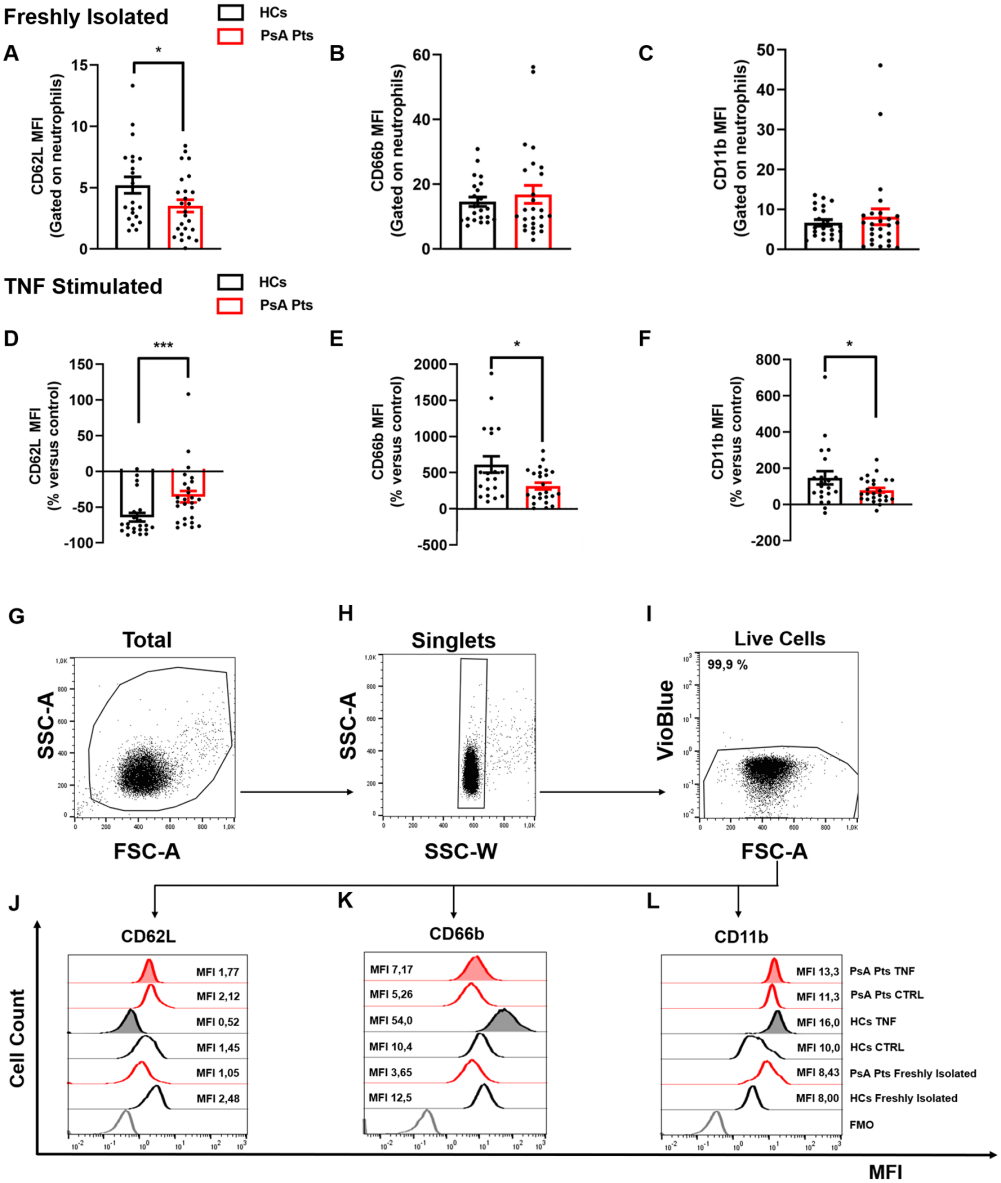
PMNs from peripheral blood of PsA patients (PsA Pts, red borders) and HCs (HCs, black borders), freshly isolated **(A–C)** and/or stimulated with TNF (10 ng/mL) **(D–F)** for 60 min at +37 °C. Cells were stained for the neutrophil activation markers CD62L **(A, D, J)**, CD66b **(B, E, K)**, and CD11b **(C, F, L)**, then subjected to cytofluorimetric analysis. Each data point in panels **(A–F)** represents one patient (PsA Pts) or one healthy control (HC). Mean fluorescence intensity (MFI) of CD62L, CD66b, and CD11b was calculated and normalized to non-stimulated cells (control medium). Results are presented as raw MFI **(A–C)** or percentage increase versus control **(D–F)** (mean ± SEM); *p < 0.05; ***p < 0.005, analyzed by Student’s-t-test or Mann-Whitney U test, depending on the distribution of the data. **(G–I)** Representative flow cytometric panels gated on live single cells, depicting forward (FSC) and side scatter (SSC) of EasySep-purified untouched PMNs **(G, H)**. Cells positive for VioBlue include both dead cells and CCR3 cells (eosinophils), were excluded using a negative gate **(I)**. Representative histograms illustrating MFI and cell count for CD62L **(J)**, CD66b **(K)**, and CD11b **(L)** on peripheral blood PMNs from HCs and PsA patients (PsA Pts), both non-stimulated (CTRL) and stimulated with TNF. MFI, Mean fluorescence intensity; FMO, Fluorescence minus one.

The flow cytometry gating strategy used to identify PMNs from peripheral blood samples of HCs and PsA patients was based on their size and granularity. Flow cytometric panels were gated on live single cells, displaying forward scatter (FSC) and side scatter (SSC) for EasySep-purified untouched PMNs. VioBlue-positive cells, which included both dead cells and CCR3-positive cells (eosinophils), were excluded based on a negative gate. Mean Fluorescence Intensity (MFI) measurements of CD66b, CD11b, and CD62L were used to assess the brightness and the abundance of these antigens on the surface of PMNs from HCs and PsA patients. Fluorescence Minus One (FMO) controls included: an unstained sample; a sample with Zombie Violet dye only (BioLegend, San Diego, CA, USA); a sample with APC-conjugated anti-CD66b and VioBlue-conjugated anti-CD193 (CCR3) monoclonal antibodies (Miltenyi Biotec, Bergisch Gladbach, Germany); a sample with anti-CD66b, anti-CD193, and PerCP-conjugated anti-CD11b monoclonal antibodies (Miltenyi Biotec, Bergisch Gladbach, Germany); and a sample stained with anti-CD66b, anti-CD193, anti-CD11b and FITC-conjugated anti-CD62L monoclonal antibodies (Miltenyi Biotec, Bergisch Gladbach, Germany). Representative flow cytometric panels illustrating the complete gating strategy for HCs and PsA patients are shown in **Figures 1G–I**.

### ROS production

PMNs (2 × 10^6^ cells/mL) were resuspended in RPMI 1640 medium supplemented with 10% FBS and antibiotics for 30 min at +37°C. Following incubation, cells were washed with PBS and incubated for 30 min with 2’,7’-dichlorodihydrofluorescein diacetate (H_2_DCF-DA; 10 µg/mL; Life Technologies, Carlsbad, CA, USA). PMNs were then washed with PBS and resuspended in RPMI with 5% FBS and various immunologic [LPS (100 ng/mL; SigmaAldrich, Milan, Italy), fMLP (1 µM; SigmaAldrich, Milan, Italy), or TNF (10 ng/mL; Miltenyi Biotec, Bergisch Gladbach, Germany)] or non-immunologic [phorbol 12-myristate 13-acetate (PMA; 10 ng/mL; SigmaAldrich, Milan, Italy)] stimuli. Cells were immediately analyzed in a 96-well plate and using an EnSpire Multimode Plate Reader (Perkin Elmer, Waltham, MA, USA). The MFI of DCF was measured at an excitation wavelength of 492–495 nm and an emission wavelength of 517–527 nm. All experiments were conducted in duplicate.

### Neutrophil phagocytic activity

To assess phagocytosis, 1.5 × 10^5^ PMNs were seeded into 96-well plate (Thermo Fisher Scientific, Waltham, MA, USA) and stimulated with PMA (10 ng/mL), LPS (100 ng/mL), fMLP (1 µM), TNF (10 ng/mL), or control medium (RPMI 1640 medium with 5% FBS; Euroclone, Milan, Italy) as negative control for 60 min at +37°C. Following stimulation, cells were washed with PBS, resuspended in RPMI 1640 medium supplemented with 5% FBS, and incubated with pHrodo™ Green *E. coli* BioParticles^®^ conjugate (100 µg/mL; Life Technologies, Carlsbad, CA, USA) for 60 min at +37°C in the presence of cytochalasin B (10 µM; SigmaAldrich, Milan, Italy) to inhibit phagocytosis. Phagocytosis was evaluated by acquiring samples on the MACS Quant Analyzer 10 (Miltenyi Biotec, Bergisch Gladbach, Germany) and data were analyzed by FlowJo software (v.10). All experiments were performed in duplicate.

### Quantification of neutrophil-related mediators

The concentrations of MMP-9, MPO, TNF, IL-17, and IL-23, in cell-free conditioned media (CM) and patient sera were determined using commercially available ELISA kits (R&D Systems, Minneapolis, MN, USA). The absorbance of the samples was measured at 450 nm using a microplate reader (Tecan, Grödig, Austria). The ELISA detection ranges were 31.2 to 2000 pg/mL (MMP-9), 62.5 to 4000 pg/mL (MPO), 15.6 to 1000 pg/mL (TNF and IL-17), and 125.0 to 8000 pg/mL (IL-23). Data analysis was performed using Microsoft Excel 2019 software (Microsoft Corporation, Redmond, WA, USA), with subsequent statistical analysis conducted using GraphPad Prism 8 (GraphPad Software, La Jolla, CA, USA). All experiments were performed in duplicate.

### Quantification of NET biomarkers in cell-free supernatant

Free DNA (dsDNA), a marker for NET formation (21, 22), was measured using a Quant-iT™ PicoGreen dsDNA Assay Kit (Thermo Fisher Scientific, Waltham, MA, USA) according to the manufacturer’s instructions. The concentration of MPO-DNA complexes in PMN supernatants from PsA patients and HCs was measured as previously described (23). Briefly, 96-well microplates (Thermo Fisher Scientific, Waltham, MA, USA) were coated overnight at +4°C with the monoclonal mouse anti-human MPO antibody (5 mg/mL; Bio-Rad, CA, USA) diluted in PBS. After blocking with 1% bovine serum albumin (BSA) in PBS, samples and then anti-DNA-POD antibodies from the Cell Death Detection ELISA kit (Roche, Basel, Swiss) were added sequentially to the wells and incubated for 2 hours each at +22°C. Following incubation, substrate solution from the Cell Death Detection ELISA kit (Roche, Basel, Swiss) was added, and absorbance was measured at 405 nm using a microplate reader (Tecan, Grödig, Austria, GmbH). PMNs (1.5 × 10^5^ cells) were seeded in a 96-well plate and stimulated with LPS (100 ng/mL), fMLP (1 µM), TNF (10 ng/mL), PMA (10 ng/mL) or control medium (RPMI 1640 medium with 5% FBS; Euroclone, Milan, Italy), for 60 min at +37°C. Supernatants from stimulated PMNs were collected after 60 min and stored at -80°C until analysis. The absorbance of dsDNA was measured with an excitation wavelength of 480 nm and an emission wavelength of 520 nm using the EnSpire Multimode Plate Reader (Perkin Elmer, Waltham, MA, USA). MPO-DNA complex absorbance was measured at 405 nm with a microplate reader (Tecan, Grödig, Austria). The ELISA sensitivity range for dsDNA was 25 pg/mL–1000 ng/mL. All experiments were performed in duplicate.

### Serum NET biomarker detection

The concentration of CitH3, a specific NET biomarker (24), was measured in the sera of PsA patients and HCs using an ELISA kit from Cayman Chemicals (Ann Arbor, MI, USA) according to the manufacturer’s instructions. This assay employed a specific monoclonal antibody for histone H3 citrullinated at residue R2, R8, and R17 (clone 11D3). Absorbance at 450 nm was determined using a microplate reader (Tecan, Grödig, Austria). The ELISA sensitivity range was 0.15–10 ng/mL. All experiments were performed in duplicate.

### Statistical analysis

Statistical analyses were performed using GraphPad Prism 8 (GraphPad Software, La Jolla, CA, USA). Results are presented as mean ± SEM of the specified number of experiments. Data normality was assessed using the D’Agostino & Pearson normality test. If data were normally distributed at a 0.05 significance level, parametric tests were applied. For non-normally distributed data, nonparametric tests were used. Group comparisons were made using Student’s t-test or Mann–Whitney U test, depending on the parametric or nonparametric distribution of the continuous variables. Repeated measures one-way or two-way ANOVA were used where appropriate, as described in the figure legends. Correlations between two variables were assessed using Pearson and/or Spearman rank correlation analysis and reported as the coefficient of correlation (r). Statistical significance was set at *p* < 0.05.

## Results

### Patients

In this study, 31 PsA patients and 22 HCs, matched for sex and age, were recruited at the Division of Allergy and Clinical Immunology of the University of Naples Federico II (Naples, Italy). Eligible patients were aged 18–70 years and had a diagnosis of PsA according to the ClASsification criteria for Psoriatic ARthritis (CASPAR). All patients presented with peripheral joint involvement, and 32.3% (10/31) also exhibited radiologically evident axial involvement. Of the 31 patients, 87.1% displayed cutaneous psoriasis (27/31), and 71% (22/31) had onychopathy. Among the enrolled patients, 45.2% were treatment-naïve, 22.6% were receiving low-dose oral corticosteroids (OCS), with or without disease-modifying antirheumatic drugs (DMARDs), and 29% were on DMARD therapy (methotrexate). One patient was on a combination of methotrexate and biological therapy (adalimumab). The main clinicopathological features of PsA patients and HCs are summarized in **Table 1**.

**Table 1:** Clinicopathological features of PsA patients (N=31) and HCs (N=22).

Characteristics	PsA Patients (N, %)	HCs (N, %)
Age (years)		
Mean ± SD	57.8 ± 11.7	47.9 ± 14.6
Sex		
Male	8 (25.8)	8 (36.3)
Female	23 (74.2)	14 (63.6)
BMI (kg/m²)		
18.0–24.9	10 (32.3)	9 (40.9)
25.0–29.9	8 (25.8)	9 (40.9)
>30–Obesity	13 (41.9)	4 (18.1)
Current or former smoker		
Yes	19 (61.3)	16 (72.7)
No	12 (38.7)	6 (27.2)
Comorbidities:		
Cardiovascular		
Yes	16 (51.6)	5 (22.7)
No	15 (48.4)	17 (77.2)
Metabolic disorders		
Yes	24 (77.4)	11 (50.0)
No	7 (22.6)	11 (50.0)
Neurologic disease		
Yes	5 (16.1)	0 (0)
No	26 (83.9)	22 (100)
Psychiatric illness		
Yes	12 (38.7)	1 (4.55)
No	19 (61.3)	21 (95.4)
Thyroid disease		
Yes	15 (48.4)	3 (13.6)
No	16 (51.6)	19 (86.3)
Age at onset of symptoms (years)		
Mean ± SD	43.7 ± 12.9	N/A
PsA subtypes		
Peripheral joint involvement	31 (100.0)	N/A
Axial involvement	10 (32.3)	N/A
Evidence of psoriasis	27 (87.1)	N/A
Onychopathy	22 (71.0)	N/A
Dactylitis	14 (45.2)	N/A
DAPSA		
Mean ± SD	26.1 ± 12.7	N/A
PASI		
Mean ± SD	0.72 ± 1.05	N/A

N/A, Not Available.

### Neutrophil activation in PsA patients

To determine the activation status of PMNs in PsA patients, we utilized flow cytometry to evaluate the expression of three activation markers: CD66b, CD11b, and CD62L (L-selectin) (25–28). The mean absolute number of peripheral blood neutrophils in PsA patients was found to be within the normal range (4561 cells/mL ± 1896 cells/mL). Freshly isolated PMNs and those stimulated with PMA (positive control), LPS, fMLP, TNF, or control medium, were stained with antibodies against CD62L, CD66b, and CD11b, then analyzed by flow cytometry (27). Freshly isolated PMNs from PsA patients showed lower expression of CD62L compared to PMNs from HCs (**Figures 1A, J**). No differences were observed in CD66b and CD11b expression (**Figures 1B–C, K–L**). Interestingly, after incubation with TNF, PMNs purified from PsA patients reduced CD62L expression, but to a lesser extent compared to HCs (**Figures 1D, J**). Similar results were obtained under LPS and fMLP stimulation (**Supplementary Figure 2C–D**). No difference in CD62L expression was detected when PMNs of HCs and PsA patients were treated with control medium or PMA (**Supplementary Figure 2A, B**). In basal conditions, PMNs from both HCs and PsA patients showed minimal expression of CD66b and CD11b, which rapidly increased after incubation with TNF compared to control medium. However, under TNF stimulation, PMNs from PsA patients increased CD66b and CD11b expression to a lesser extent compared to HCs (**Figures 1E-F, K–L**). In contrast, no significant difference was found between PsA patients and HCs in the expression of CD66b and CD11b after stimulation with control medium, PMA, LPS, and fMLP (**Supplementary Figure 2E–L**). Collectively these data indicate that freshly isolated PMNs in PsA patients are activated compared to their HC counterparts and, selectively under TNF stimulation, PMNs undergo activation (CD66b and CD11b upregulation and CD62L downregulation) to a lesser extent compared to PMNs purified from HCs.

### ROS production and phagocytic activity of PMNs in PsA patients

**Figure 2:**
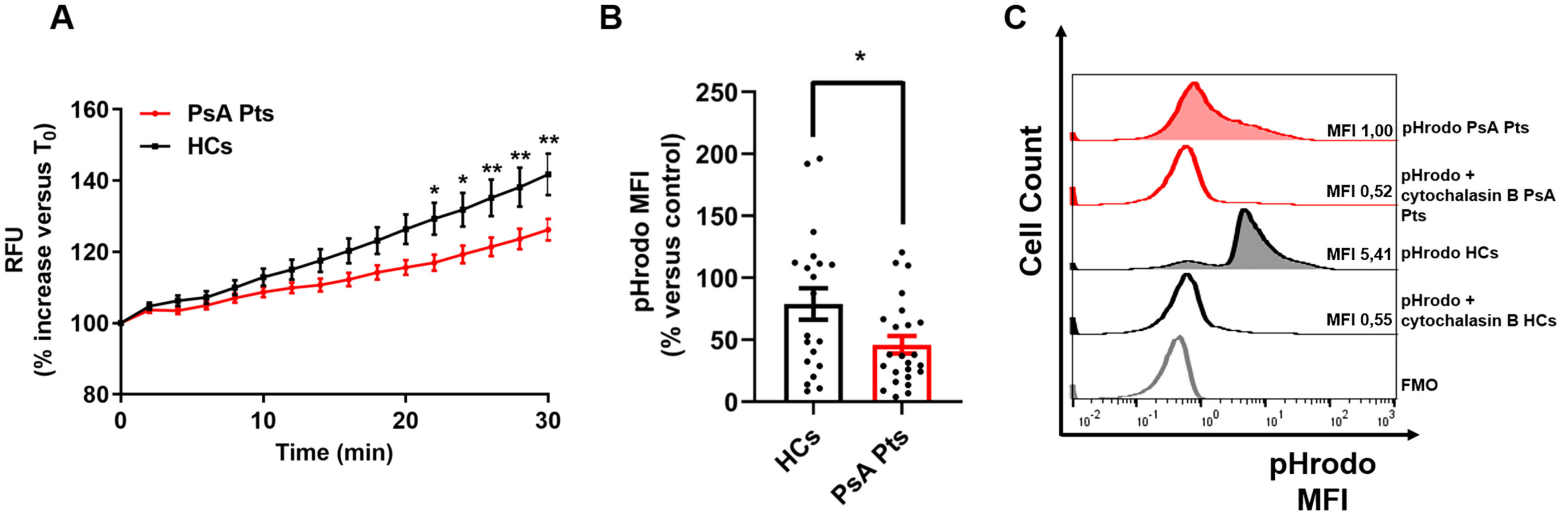
PMNs from peripheral blood of PsA patients (red lines) and HCs (black lines) were incubated with 2’,7’-dichlorodihydrofluorescein diacetate (H_2_DCFDA, 10 µM), washed, then stimulated with TNF (10 ng/mL) or control medium. Immediately following stimulation, PMNs were analyzed with a multimode microplate reader (EnSpire Multimode Plate reader, PerkinElmer), and DCF fluorescence was measured for 30 min at 2 min intervals. The results are expressed as Relative Fluorescence Unit (RFU) and percentage increase *versus* time 0 (mean ± SEM); *p < 0.05; **p < 0.01, analyzed by two-way ANOVA and Bonferroni post-test **(A)**. PMNs from peripheral blood of PsA patients (PsA Pts, red border) and HCs (HCs, black border) were stimulated with TNF (10 ng/mL) for 60 min at +37 °C, then incubated with pHrodo^TM^ Green *E*. *coli* BioParticles® conjugate (100 µg/mL) for 60 min at +37 °C, with or without cytochalasin B (as inhibitor of phagocytosis, 10 µM), followed by flow cytometric analysis. Each point represents one patient (PsA Pts) or one healthy control (HCs). MFI of cells positive for pHrodo^TM^ was calculated and normalized to the unstimulated cells (control medium). Results were expressed as percentage increase *versus* control (mean ± SEM); *p < 0.05, analyzed by Student’s t-test **(B)**. Representative histograms illustrating MFI and cell count for pHrodo assay data of peripheral blood PMNs from HCs and PsA patients (PsA Pts), in the presence or absence of cytochalasin B, after stimulation with TNF **(C)**. MFI, Mean fluorescence intensity; FMO, Fluorescence minus one.

To investigate ROS production, PMNs from PsA patients and HCs were stimulated with PMA, LPS, fMLP, and TNF, then subjected to an H_2_DCF-DA ROS detection assay. No significant difference in ROS production was observed between patients and HCs in the control medium (**Supplementary Figure 3A**). However, under TNF stimulation, PMNs isolated from PsA patients exhibited increased ROS production, although to a lesser extent compared to PMNs from HCs (**Figure 2A**). Similar trends were observed with LPS and fMLP stimulation (**Supplementary Figures 3C–D**). Following TNF stimulation, ROS production in PMNs from PsA patients and HCs became evident after 20 minutes, whereas LPS and fMLP-induced ROS production was detectable after 25 minutes. No significant difference in ROS production was detected between patients and HCs when PMNs were stimulated with PMA (**Supplementary Figure 3B**). Following stimulation with PMA, LPS, fMLP, and TNF, the phagocytic ability of PMNs from PsA patients was assessed by incubating the cells with pHrodo™ Green *E. coli* BioParticles^®^ in the presence or absence of cytochalasin B, a phagocytosis inhibitor (29). Compared to HCs, PMNs from PsA patients exhibited a significant reduction in phagocytic ability upon TNF stimulation (**Figures 2B–C**). No differences in phagocytic activity were found between PMNs from PsA patients and HCs in basal conditions or following LPS, fMLP, or PMA stimulation (**Supplementary Figures 3F–I**). These findings indicate that PMNs from PsA patients, upon stimulation with TNF, display a reduced rate of ROS production and phagocytic activity compared to HCs.

### NET biomarker release and neutrophil degranulation in PsA patients

**Figure 3:**
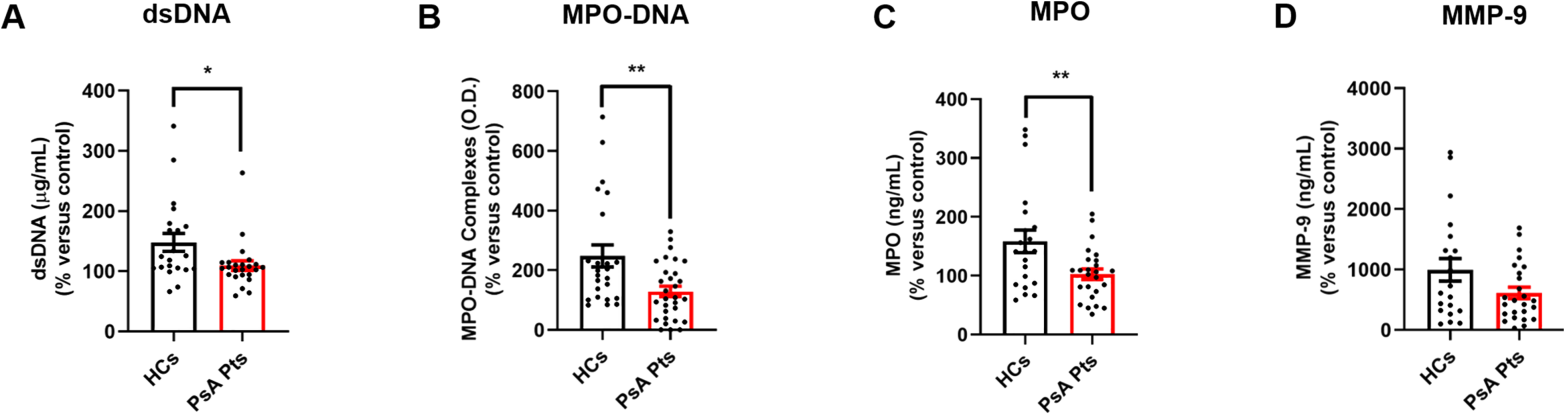
PMNs from peripheral blood of PsA patients (PsA Pts, red borders) and HCs (HCs, black borders) were cultured for 60 min at +37 °C in the presence or absence of TNF (10 ng/mL). The extracellular levels of dsDNA **(A)**, MPO-DNA complexes **(B)**, MPO **(C)**, and MMP-9 **(D)** were measured by Quant-iT^TM^ PicoGreen^TM^ dsDNA Assay Kit or by ELISA, respectively. Each point in graphs **(A–D)** represents one patient (PsA Pts) or one healthy control (HCs). The extracellular levels of dsDNA, MPO-DNA complexes, MPO, and MMP-9 were calculated and normalized to unstimulated cells (control medium; mean ± SEM); *p < 0.05; **p < 0.01; *p* for MMP-9 = 0.06, analyzed by Student’s-t-test or Mann-Whitney U test, depending on the distribution of the data.

PMNs from HCs and PsA patients were cultured in the presence of PMA, LPS, fMLP, TNF, or control medium for 60 min. After incubation, supernatants were collected to evaluate the extracellular levels of NET biomarkers (dsDNA and MPO-DNA complexes), MPO, and MMP-9. In control medium, PsA PMNs displayed reduced levels of MPO-DNA complexes in the extracellular milieu (**Supplementary Figure 4E**), while no differences in the release of dsDNA, MPO, and MMP-9 were observed (**Supplementary Figures 4A, I, M**). Upon stimulation with TNF, PMNs from PsA patients released NETs and MPO into the extracellular milieu, although lower levels than PMNs from HCs (**Figures 3A–C**). A similar trend was observed for MMP-9 release (**Figure 3D**). Similarly, under fMLP stimulation, PMNs from PsA patients released less MPO-DNA complexes than their HC counterparts (**Supplementary Figure 4H**). No differences were detected in the release of dsDNA (**Supplementary Figures 4A–D**), MPO-DNA complexes (**Supplementary Figures 4F, G**), MPO (**Supplementary Figure 4I–L**), or MMP-9 (**Supplementary Figure 4M–P**) under LPS, fMLP, or PMA stimulation. These results suggest that PMNs from PsA patients, when incubated with TNF, release lower quantities of granular enzymes (MPO and MMP-9) and NET biomarkers compared with PMNs from HCs.

### Serum levels of neutrophil-related mediators and NET biomarkers in PsA patients

**Figure 4:**
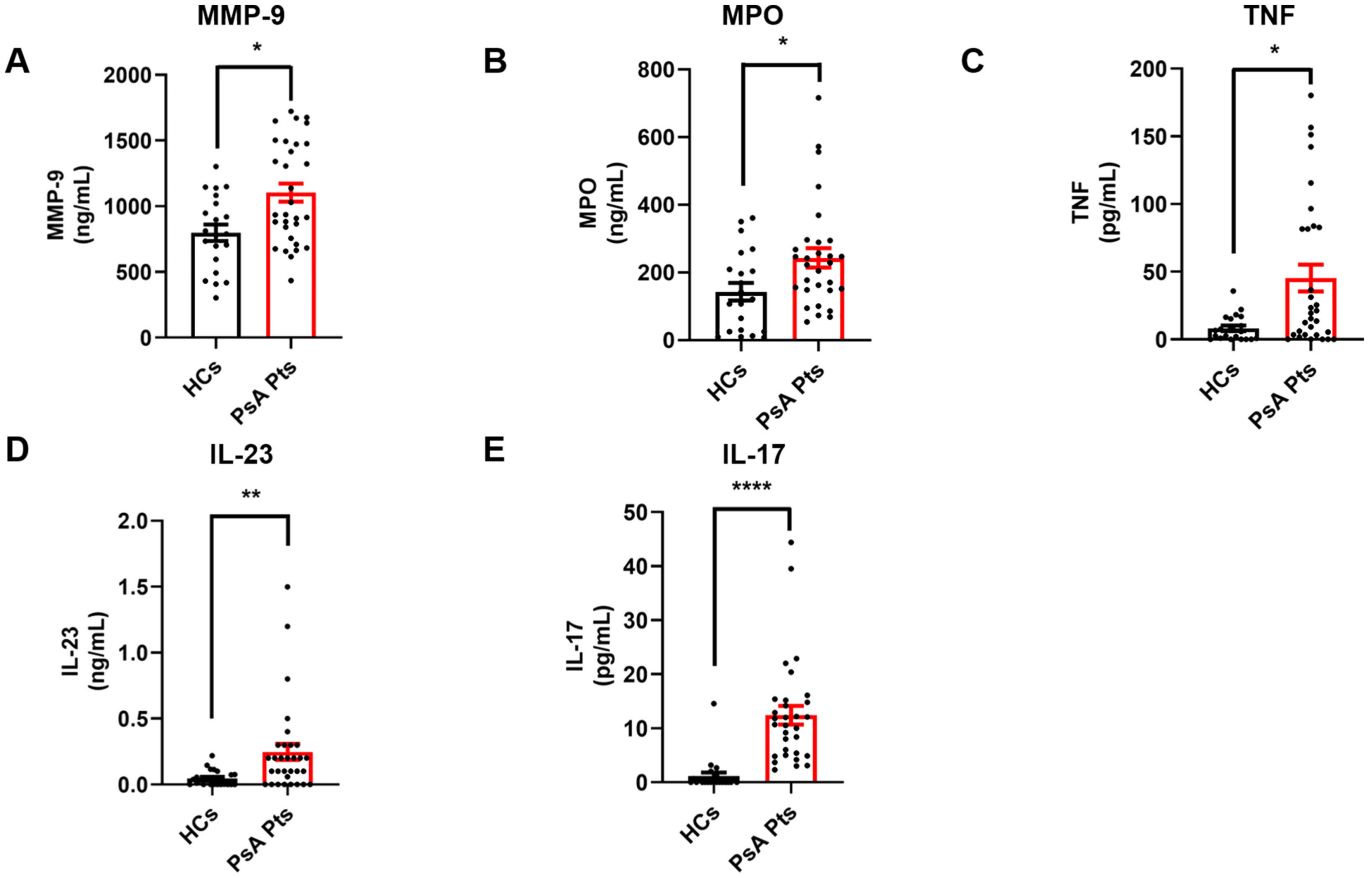
Serum concentrations of MMP-9 **(A)**, MPO **(B)**, TNF **(C)**, IL-23 **(D)**, and IL-17 **(E)** in PsA patients (PsA PTs, red borders) and HCs (HCs, black borders) were measured by ELISA. Each point in graphs **(A–E)** represents one patient (PsA Pts) or one healthy control (HCs). Results were expressed as mean ± SEM; *p < 0.05; **p < 0.01; **** *p* < 0.001. Student’s t-test or Mann-Whitney U test was employed according to the distribution of the data.

**Figure 5:**
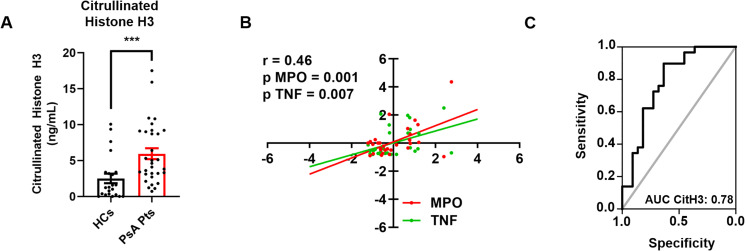
Serum concentrations of CitH3 in PsA patients (PsA PTs, red borders) and HCs (HCs, black borders) were measured by Citrullinated Histone H3 (clone11D3) ELISA. Each point represents one patient (PsA Pts) or one healthy control (HCs). Results were expressed as mean ± SEM; *** *p* < 0.005, analyzed by Student’s t-test or Mann-Whitney U test, depending on the distribution of the data **(A)**. Multiple linear regression analysis between serum concentrations of CitH3, MPO (red line), and TNF (green line) in PsA patients, with CitH3 as the dependent variable. *r^2^* = 0.46; *versus* MPO *p* = 0.0001; *versus* TNF *p* = 0.0007 **(B)**. ROC curve analysis of serum concentrations of CitH3 to evaluate the accuracy of CitH3 as a diagnostic biomarker for PsA. Area Under the Curve (AUC) = 0.78; Cut-Off = 1.65; Sensitivity = 0.90; Specificity = 0.64; CI = 2.51–7.07 **(C)**.

Inflammatory cytokines, including TNF, IL-23, and IL-17, play a crucial role in the initiation and progression of inflammatory processes in PsA (30). Serum levels of the neutrophil-related mediators MMP-9 (**Figure 4A**), MPO (**Figure 4B**), TNF (**Figure 4C**), IL-23 (**Figure 4D**), and IL-17 (**Figure 4E**) were increased in PsA patients compared to HCs. Serum concentrations of CitH3, a specific biomarker for NET identification, were also elevated in PsA patients compared to HCs (**Figure 5A**). Interestingly, circulating levels of CitH3 displayed a moderate correlation with serum concentrations of MPO and TNF (**Figure 5B**). Importantly, ROC curve analysis demonstrated that serum levels of CitH3 could accurately distinguish PsA patients from HCs with high specificity and sensitivity (**Figure 5C**). Specifically, ROC curve analysis for CitH3 showed a cut-off value of 1.65 and a confidence interval (CI) of 2.51–7.07, with sensitivity and specificity values of 0.90 and 0.64, respectively. In addition, a moderate correlation was found between serum levels of IL-23 and IL-17 (**Supplementary Figure 5**), two well-established inflammatory cytokines involved in neutrophil-related PsA pathogenesis (31). Altogether, these data indicate that neutrophils exhibit a distinct phenotype in PsA patients, suggesting that PMNs are activated under resting conditions in PsA.

## Discussion

In this study, we found that PMNs from the peripheral blood of PsA patients exhibit several distinct phenotypic and functional deficits compared to their counterparts from HCs. Specifically, upon stimulation with TNF, PMNs from PsA patients displayed lower expression of activation markers (CD66b, CD11b, and CD62L), reduced ROS production, and diminished ability to phagocytose bacterial bioparticles, compared to PMNs purified from the peripheral blood of HCs. In addition, PMNs from PsA patients released less granular enzymes (MPO and MMP-9) and NET biomarkers than those from HCs. Finally, increased levels of neutrophil-related mediators MMP-9, MPO, TNF, IL-23, and IL-17, as well as elevated levels of CitH3, a specific NET biomarker, were found in PsA patients compared to HCs.

In psoriasis, PMNs are among the first cells to migrate into psoriatic lesions (32). PMN accumulation in the dermis and epidermis results in the characteristic Kogoj pustules and Munro microabscesses (33). However, among the immune cells involved in the pathogenesis of PsA, the role of PMNs has been investigated in relatively few studies. The neutrophil-to-lymphocyte ratio has been proposed as an inflammatory parameter, correlating with disease score and response to biological therapy (34–36). Only one study investigated the circulating levels of NET biomarkers in PsA patients. Serum MPO-DNA complex levels were significantly increased in patients with PsA/PsO compared to HCs and were positively associated with the DAPSA score (9). For the first time, this study systematically investigated the *ex vivo* and *in vitro* behavior of PMNs from PsA patients from a functional perspective. In addition, we evaluated neutrophil-related mediators and circulating NETs as biomarkers of disease.

As shown in **Figure 1**, we evaluated the activation status of PMNs in PsA patients and HCs, including both freshly isolated PMNs (panels A–C) and those stimulated with TNF (panels D–F). Specifically, to investigate PMN functional behavior *in vitro*, we evaluated the changes in the expression of CD11b, CD66b, and CD62L induced by TNF in PsA patients and controls. Under basal conditions, PsA PMNs expressed lower levels of CD62L compared to HCs, while there was no difference between PsA patients and HCs in the expression of CD66b and CD11b, which are minimally expressed on the cell surface (**Figures 1B, C**) (37). *Ex vivo*, after stimulation with TNF, PMNs increased the expression of CD66b (**Figure 1E**) and CD11b (**Figure 1F**), with a more pronounced increase in HCs compared to PsA patients. Conversely, CD62L, normally expressed on the surface of resting PMNs, is rapidly downregulated after cell activation (27). In PsA patients, TNF stimulation led to CD62L downregulation in PMNs, albeit to a lesser extent than in HCs (**Figure 1D**). Based on the current understanding of PMN activation dynamics, it is widely demonstrated that *in vitro* activation with various stimuli leads to CD62L shedding (38). Consistent with this, our findings of CD62L downregulation under TNF likely result from shedding. Collectively, these findings support the hypothesis that neutrophils from PsA patients are presumably desensitized *in vivo* and less responsive to *in vitro* activation by TNF.

ROS play multiple and significant roles in the pathogenesis of several inflammatory disorders (39), including PsO and PsA (40). Neutrophil ROS production can be induced by several stimuli. LPS and fMLP activate ROS production upon the engagement of toll-like receptor 4 (TLR4) (41) and formyl peptide receptors (42), respectively. TNF is also capable of enhancing the neutrophil respiratory burst (43, 44). As displayed in **Figure 2A**, we found that PMNs from PsA patients produced reduced ROS compared to HCs when stimulated with TNF. In addition, after stimulation with TNF, PMNs from PsA patients exhibited reduced phagocytic capability compared to those from HCs (**Figures 2B, C**), which is one of the microbicidal mechanisms utilized by PMNs to eliminate microorganisms (45). These data further confirm the hypothesis that PMNs from PsA patients are exhausted *in vitro*. Importantly, ROS acts both as a regulator of inflammation (46) and as a suppressor of arthritis and encephalomyelitis (47–49). Although ROS has been suggested to be pathogenic in PsO (50), a murine model of imiquimod-induced psoriasis demonstrated that the disease was attenuated with elevated ROS levels and aggravated in with reduced ROS levels (51). In mannan-induced inflammation, ROS deficiency promoted PsO and arthritis, which were suppressed by monocyte/macrophage-derived ROS, similar to collagen-induced arthritic inflammation (49). However, the exact mechanism by which ROS protects against PsO and PsA requires further detailed investigation. More broadly, the hypo-responsiveness of neutrophils *in vivo* under TNF stimulation can be interpreted in two different ways. One hypothesis is that neutrophils are exhausted *in vivo* and thus unable to respond further to inflammatory stimuli. Alternatively, reduced neutrophil activation might represent a self-limiting mechanism to mitigate TNF-driven inflammation, thereby reducing overall patient injury. Further experiments are necessary to elucidate these mechanisms.

Human PMNs secrete a variety of pro-inflammatory mediators and cytokines, as well as NETs (52). The role of TNF in NET release has been documented in PMNs from patients suffering from inflammatory diseases (53). In our study, we investigated the presence of NETs in the supernatants of PMNs isolated from PsA patients (**Figure 3**). Quantification of circulating NETs can be performed using various analytical methods; however, a standardized assay specifically for NETs remains unavailable. Therefore, studies typically measure NET biomarkers such as free DNA (dsDNA), nucleosomes, circulating CitH3, and NET-associated proteins (e.g., NE, MPO). While MPO and NE are components and putative biomarkers of NETs, they are not specific (54), as they can be released during neutrophil degranulation independently of NET formation (55). Conjugates such as MPO-DNA complexes or CitH3 may offer greater specificity compared to dsDNA alone (56, 57). To evaluate *in vitro* NET release, we measured two NET biomarkers: dsDNA (58) and the more specific MPO-DNA complexes (9, 56, 58). Our findings revealed that PMNs from PsA patients displayed reduced levels of NET biomarkers in the extracellular milieu (**Figures 3A–B**) upon stimulation with TNF, compared to PMNs from HCs. Additionally, MMP-9 plays a key role in the development of inflammatory disorders (e.g. rheumatoid arthritis; RA) (59). MPO is a cationic heme-containing enzyme stored in the primary/azurophilic granules of PMNs, involved in ROS and NET production pathways, as well as in several inflammatory diseases (60). We found that PMNs from PsA patients exhibited a reduced release of granular enzymes, specifically MPO (**Figure 3C**) and MMP-9 (**Figure 3D**), upon TNF stimulation compared to PMNs isolated from the peripheral blood of HCs.

In subsequent experiments, we investigated the circulating levels of neutrophil-related mediators (**Figure 4**). We found higher serum concentrations of neutrophil-associated factors (MMP-9, MPO, TNF, IL-23, and IL-17; **Figures 4A–E**) in PsA patients compared to controls. These results align with findings in patients with RA, where elevated MMP-9 and MPO levels have been reported in the plasma and synovial fluid (59, 61). In addition, patients with PsA and PsO have elevated levels of TNF in their joint tissues and lesional skin (62). The IL-23/IL-17 pathway plays a primary pathogenic role in psoriatic disorders, and IL-23/IL-17 targeted therapies have demonstrated efficacy in psoriasis (63, 64). Notably, in PsA patients who responded to anti-IL-17 therapy, RNA sequencing revealed transcriptomic changes predictive of downregulation in cytokine signaling and chemotaxis pathways, alongside upregulation of *de novo* gene expression pathways, including translation initiation, mRNA catabolism, and translation in peripheral blood neutrophils. These results suggest significant alterations in neutrophil properties following secukinumab treatment in PsA (65). Similarly, anti-IL-23 therapy led to a more pronounced downregulation of genes associated with neutrophil and macrophage gene sets in responders than in non-responders (66). We also found a positive correlation between IL-17 and IL-23, both of which are commonly associated with PMNs and PsA (67, 68), supporting their involvement in this inflammatory condition. Interestingly, in a murine model of mannan-induced PsA, uncontrolled macrophage activation triggered TNF-mediated activation of γδ T cells in the skin, leading to localized IL-17A production, which subsequently promoted neutrophil infiltration and activation (69). Further longitudinal studies evaluating the impact of anti-TNF/IL-17/IL-23 blocking therapies on neutrophil functions could provide additional insights into these mechanisms.

Finally, we investigated the circulating levels of NET biomarkers in the sera of PsA patients and HCs (**Figure 5**). Given that ELISA detection of MPO-DNA complexes *in vivo* is error-prone and yields limited information (70), we validated our findings by measuring serum levels of CitH3, which is considered a more reliable biomarker of NET formation (54). We found elevated circulating levels of CitH3 in PsA patients compared to HCs (**Figure 5A**) and identified a positive correlation between serum levels of CitH3, MPO, and TNF (**Figure 5B**). These findings corroborate the established association between MPO and NET release, as MPO is typically released during the NETosis process (71). In addition, the correlation with TNF levels suggests an interplay between the overall inflammatory condition in PsA and NETs (72). Indeed, recent evidence highlights that NETs are released when PMNs are activated and play critical roles in the onset and persistence of inflammation and autoimmune responses (73). Importantly, ROC curve analysis demonstrated that circulating levels of CitH3 could serve as a potential biomarker for distinguishing PsA patients from healthy individuals (**Figure 5C**). These results align with previous evidence demonstrating the diagnostic potential of cell-free nucleosomes for differentiating active *versus* non-active axial spondyloarthritis (74) and RA (75). Collectively, our results demonstrate that PsA patients display higher basal levels of neutrophil-related mediators and NET biomarkers compared to HCs, confirming the distinct neutrophil signature associated with PsA.

Our data demonstrate that PMNs from PsA patients, upon TNF stimulation, exhibit diminished efficiency in several key effector functions, including activation, ROS production, phagocytosis of bacterial particles, and the release of granular enzymes and NETs. This impaired behavior may be attributed to the heightened inflammatory milieu characteristic of PsA, which could contribute to a state of “pre-activation” in these cells, leading to reduced reactivity during subsequent *in vitro* stimulation. This phenomenon, known as “innate immune tolerance”, occurs when cells exposed to persistent inflammatory stimuli become incapable of effectively activating gene transcription and performing their normal functions upon restimulation (76). Such functional deficits in neutrophils from PsA patients may contribute to ongoing inflammatory processes and could potentially impair the role of PMNs in the host defense.

This study has certain limitations. The sample size was relatively small, and while our findings suggest an exhausted phenotype of neutrophils, larger and multi-center studies are required to further elucidate the role of innate immunity in PsA and validate the existence of neutrophil tolerance in these patients. In addition, subgroup analyses stratified by different medications were not conducted, leaving the potential impact of various conventional and biologic DMARDs on neutrophil phenotype unexplored.

Together, our findings underscore the need for a reassessment of PMNs and neutrophil-related mediators in the pathophysiology of PsA. Further investigations involving larger cohorts of both healthy donors and patients could corroborate and better highlight the role of PMNs and neutrophil-related mediators in PsA.

## Data Availability

The raw data supporting the conclusions of this article will be made available by the authors, without undue reservation.

## References

[1] Azuaga AB Ramirez J Canete JD. Psoriatic arthritis: pathogenesis and targeted therapies. Int J Mol Sci. (2023) 24. doi: 10.3390/ijms240549014901 PMC10003101 36902329

[2] Ritchlin CT Colbert RA Gladman DD. Psoriatic arthritis. N Engl J Med. (2017) 376:2095–6. doi: 10.1056/NEJMc1704342 28538114

[3] Villani AP Rouzaud M Sevrain M Barnetche T Paul C Richard MA. Prevalence of undiagnosed psoriatic arthritis among psoriasis patients: Systematic review and meta-analysis. J Am Acad Dermatol. (2015) 73:242–8. doi: 10.1016/j.jaad.2015.05.001 26054432

[4] Perez-Chada LM Merola JF. Comorbidities associated with psoriatic arthritis: Review and update. Clin Immunol. (2020) 214:108397. doi: 10.1016/j.clim.2020.108397 32229290

[5] O’Brien-Gore C Gray EH Durham LE Taams LS Kirkham BW. Drivers of inflammation in psoriatic arthritis: the old and the new. Curr Rheumatol Rep. (2021) 23:40. doi: 10.1007/s11926-021-01005-x 33909160

[6] Jeljeli MM Adamopoulos IE. Innate immune memory in inflammatory arthritis. Nat Rev Rheumatol. (2023) 19:627–39. doi: 10.1038/s41584-023-01009-0 PMC10721491 37674048

[7] Mormile I Rossi FW Prevete N Granata F Pucino V de Paulis A. The N-formyl peptide receptors and rheumatoid arthritis: A dangerous liaison or confusing relationship? Front Immunol. (2021) 12:685214. doi: 10.3389/fimmu.2021.685214 34220836 PMC8253054

[8] Wang WM Jin HZ. Role of neutrophils in psoriasis. J Immunol Res. (2020) 2020:3709749. doi: 10.1155/2020/3709749 32587871 PMC7293746

[9] Li B Li G Yang X Song Z Wang Y Zhang Z. NETosis in psoriatic arthritis: serum MPO-DNA complex level correlates with its disease activity. Front Immunol. (2022) 13:911347. doi: 10.3389/fimmu.2022.911347 35774788 PMC9238436

[10] Lin AM Rubin CJ Khandpur R Wang JY Riblett M Yalavarthi S. Mast cells and neutrophils release IL-17 through extracellular trap formation in psoriasis. J Immunol. (2011) 187:490–500. doi: 10.4049/jimmunol.1100123 21606249 PMC3119764

[11] Chiang CC Cheng WJ Korinek M Lin CY Hwang TL. Neutrophils in psoriasis. Front Immunol. (2019) 10:2376. doi: 10.3389/fimmu.2019.02376 31649677 PMC6794444

[12] Yamanaka K Umezawa Y Yamagiwa A Saeki H Kondo M Gabazza EC. Biologic therapy improves psoriasis by decreasing the activity of monocytes and neutrophils. J Dermatol. (2014) 41:679–85. doi: 10.1111/1346-8138.12560 25099154

[13] Papayannopoulos V. Neutrophil extracellular traps in immunity and disease. Nat Rev Immunol. (2018) 18:134–47. doi: 10.1038/nri.2017.105 28990587

[14] Brinkmann V Reichard U Goosmann C Fauler B Uhlemann Y Weiss DS. Neutrophil extracellular traps kill bacteria. Science. (2004) 303:1532–5. doi: 10.1126/science.1092385 15001782

[15] Urban CF Reichard U Brinkmann V Zychlinsky A. Neutrophil extracellular traps capture and kill Candida albicans yeast and hyphal forms. Cell Microbiol. (2006) 8:668–76. doi: 10.1111/j.1462-5822.2005.00659.x 16548892

[16] Saitoh T Komano J Saitoh Y Misawa T Takahama M Kozaki T. Neutrophil extracellular traps mediate a host defense response to human immunodeficiency virus-1. Cell Host Microbe. (2012) 12:109–16. doi: 10.1016/j.chom.2012.05.015 22817992

[17] Raftery MJ Lalwani P Krautkrämer E Peters T Scharffetter-Kochanek K Kruger R. beta2 integrin mediates hantavirus-induced release of neutrophil extracellular traps. J Exp Med. (2014) 211:1485–97. doi: 10.1084/jem.20131092 PMC4076588 24889201

[18] Frasca L Palazzo R Chimenti MS Alivernini S Tolusso B Bui L. Anti-LL37 antibodies are present in psoriatic arthritis (PsA) patients: new biomarkers in psA. Front Immunol. (2018) 9:19361936. doi: 10.3389/fimmu.2018.019361936 PMC6154218 30279686

[19] Calzetti F Tamassia N Arruda-Silva F Gasperini S Cassatella MA. The importance of being “pure” neutrophils. J Allergy Clin Immunol. (2017) 139:352–5.e6. doi: 10.1016/j.jaci.2016.06.025 27567327

[20] Borriello F Iannone R Di Somma S Loffredo S Scamardella E Galdiero MR. GM-CSF and IL-3 modulate human monocyte TNF-alpha production and renewal in *in vitro* models of trained immunity. Front Immunol. (2016) 7:680. doi: 10.3389/fimmu.2016.00680 28138327 PMC5237654

[21] Demers M Wagner DD. NETosis: a new factor in tumor progression and cancer-associated thrombosis. Semin Thromb Hemost. (2014) 40:277–83. doi: 10.1055/s-0034-1370765 PMC4112728 24590420

[22] Varricchi G Modestino L Poto R Cristinziano L Gentile L Postiglione L. Neutrophil extracellular traps and neutrophil-derived mediators as possible biomarkers in bronchial asthma. Clin Exp Med. (2022) 22:285–300. doi: 10.1007/s10238-021-00750-8 34342773 PMC9110438

[23] Kessenbrock K Krumbholz M Schonermarck U Back W Gross WL Werb Z. Netting neutrophils in autoimmune small-vessel vasculitis. Nat Med. (2009) 15:623–5. doi: 10.1038/nm.1959 PMC2760083 19448636

[24] Grilz E Mauracher LM Posch F Konigsbrugge O Zochbauer-Muller S Marosi C. Citrullinated histone H3, a biomarker for neutrophil extracellular trap formation, predicts the risk of mortality in patients with cancer. Br J Haematol. (2019) 186:311–20. doi: 10.1111/bjh.15906 PMC6618331 30968400

[25] Condliffe AM Chilvers ER Haslett C Dransfield I. Priming differentially regulates neutrophil adhesion molecule expression/function. Immunology. (1996) 89:105–11. doi: 10.1046/j.1365-2567.1996.d01-711.x PMC1456672 8911147

[26] Stocks SC Ruchaud-Sparagano MH Kerr MA Grunert F Haslett C Dransfield I. CD66: role in the regulation of neutrophil effector function. Eur J Immunol. (1996) 26:2924–32. doi: 10.1002/eji.1830261218 8977287

[27] Modestino L Cristinziano L Trocchia M Ventrici A Capone M Madonna G. Melanoma-derived soluble mediators modulate neutrophil biological properties and the release of neutrophil extracellular traps. Cancer Immunol Immunother. (2023) 72:3363–76. doi: 10.1007/s00262-023-03493-5 PMC10491523 37525065

[28] Galdiero MR Varricchi G Loffredo S Bellevicine C Lansione T Ferrara AL. Potential involvement of neutrophils in human thyroid cancer. PloS One. (2018) 13:e0199740. doi: 10.1371/journal.pone.0199740 29953504 PMC6023126

[29] Impellizzieri D Egholm C Valaperti A Distler O Boyman O. Patients with systemic sclerosis show phenotypic and functional defects in neutrophils. Allergy. (2022) 77:1274–84. doi: 10.1111/all.15073 PMC9293168 34467524

[30] Lee BW Moon SJ. Inflammatory cytokines in psoriatic arthritis: understanding pathogenesis and implications for treatment. Int J Mol Sci. (2023) 24. doi: 10.3390/ijms241411662 PMC10381020 37511421

[31] Laan M Cui ZH Hoshino H Lotvall J Sjostrand M Gruenert DC. Neutrophil recruitment by human IL-17 via C-X-C chemokine release in the airways. J Immunol. (1999) 162:2347–52. doi: 10.4049/jimmunol.162.4.2347 9973514

[32] Murphy M Kerr P Grant-Kels JM. The histopathologic spectrum of psoriasis. Clin Dermatol. (2007) 25:524–8. doi: 10.1016/j.clindermatol.2007.08.005 18021888

[33] Schon MP Broekaert SM Erpenbeck L. Sexy again: the renaissance of neutrophils in psoriasis. Exp Dermatol. (2017) 26:305–11. doi: 10.1111/exd.13067 27194625

[34] Esen M. The effect of IL17 and IL23 inhibitors on hematological parameters and C-reactive protein in psoriasis patients. Cutan Ocul Toxicol. (2024) 43:38–45. doi: 10.1080/15569527.2023.2275020 37897439

[35] Kommoss KS Bieler T Ringen J Lehmann A Mihalceanu S Hobohm L. A simple tool for evaluation of inflammation in psoriasis: Neutrophil-to-lymphocyte and platelet-to-lymphocyte ratio as markers in psoriasis patients and related murine models of psoriasis-like skin disease. J Mol Med (Berl). (2024) 102:247–55. doi: 10.1007/s00109-023-02406-4 PMC10857970 38127137

[36] Zhang Y Qian H Kuang YH Wang Y Chen WQ Zhu W. Evaluation of the inflammatory parameters as potential biomarkers of systemic inflammation extent and the disease severity in psoriasis patients. Arch Dermatol Res. (2024) 316:229. doi: 10.1007/s00403-024-02972-8 38787405

[37] Cristinziano L Modestino L Loffredo S Varricchi G Braile M Ferrara AL. Anaplastic thyroid cancer cells induce the release of mitochondrial extracellular DNA traps by viable neutrophils. J Immunol. (2020) 204:1362–72. doi: 10.4049/jimmunol.1900543 31959732

[38] Kuhns DB Long Priel DA Gallin JI. Loss of L-selectin (CD62L) on human neutrophils following exudation. vivo. Cell Immunol. (1995) 164:306–10. doi: 10.1006/cimm.1995.1174 7544694

[39] Goud PT Bai D Abu-Soud HM. A multiple-hit hypothesis involving reactive oxygen species and myeloperoxidase explains clinical deterioration and fatality in COVID-19. Int J Biol Sci. (2021) 17:62–72. doi: 10.7150/ijbs.51811 33390833 PMC7757048

[40] Khmaladze I Nandakumar KS Holmdahl R. Reactive oxygen species in psoriasis and psoriasis arthritis: relevance to human disease. Int Arch Allergy Immunol. (2015) 166:135–49. doi: 10.1159/000375401 25824670

[41] Asehnoune K Strassheim D Mitra S Kim JY Abraham E. Involvement of reactive oxygen species in Toll-like receptor 4-dependent activation of NF-kappa B. J Immunol. (2004) 172:2522–9. doi: 10.4049/jimmunol.172.4.2522 14764725

[42] Weiss E Kretschmer D. Formyl-peptide receptors in infection, inflammation, and cancer. Trends Immunol. (2018) 39:815–29. doi: 10.1016/j.it.2018.08.005 30195466

[43] Maianski NA Roos D Kuijpers TW. Tumor necrosis factor alpha induces a caspase-independent death pathway in human neutrophils. Blood. (2003) 101:1987–95. doi: 10.1182/blood-2002-02-0522 12393608

[44] Dewas C Dang PM Gougerot-Pocidalo MA El-Benna J. TNF-alpha induces phosphorylation of p47(phox) in human neutrophils: partial phosphorylation of p47phox is a common event of priming of human neutrophils by TNF-alpha and granulocyte-macrophage colony-stimulating factor. J Immunol. (2003) 171:4392–8. doi: 10.4049/jimmunol.171.8.4392 14530365

[45] Borregaard N. Neutrophils, from marrow to microbes. Immunity. (2010) 33:657–70. doi: 10.1016/j.immuni.2010.11.011 21094463

[46] Sareila O Kelkka T Pizzolla A Hultqvist M Holmdahl R. NOX2 complex-derived ROS as immune regulators. Antioxid Redox Signal. (2011) 15:2197–208. doi: 10.1089/ars.2010.3635 20919938

[47] Hultqvist M Olofsson P Holmberg J Backstrom BT Tordsson J Holmdahl R. Enhanced autoimmunity, arthritis, and encephalomyelitis in mice with a reduced oxidative burst due to a mutation in the Ncf1 gene. Proc Natl Acad Sci U.S.A. (2004) 101:12646–51. doi: 10.1073/pnas.0403831101 PMC515111 15310853

[48] Kelkka T Hultqvist M Nandakumar KS Holmdahl R. Enhancement of antibody-induced arthritis via Toll-like receptor 2 stimulation is regulated by granulocyte reactive oxygen species. Am J Pathol. (2012) 181:141–50. doi: 10.1016/j.ajpath.2012.03.031 22642907

[49] Gelderman KA Hultqvist M Pizzolla A Zhao M Nandakumar KS Mattsson R. Macrophages suppress T cell responses and arthritis development in mice by producing reactive oxygen species. J Clin Invest. (2007) 117:3020–8. doi: 10.1172/JCI31935 PMC1994618 17909630

[50] Zhou Q Mrowietz U Rostami-Yazdi M. Oxidative stress in the pathogenesis of psoriasis. Free Radic Biol Med. (2009) 47:891–905. doi: 10.1016/j.freeradbiomed.2009.06.033 19577640

[51] Kim HR Lee A Choi EJ Hong MP Kie JH Lim W. Reactive oxygen species prevent imiquimod-induced psoriatic dermatitis through enhancing regulatory T cell function. PloS One. (2014) 9:e91146. doi: 10.1371/journal.pone.0091146 24608112 PMC3946742

[52] Cassatella MA Ostberg NK Tamassia N Soehnlein O. Biological roles of neutrophil-derived granule proteins and cytokines. Trends Immunol. (2019) 40:648–64. doi: 10.1016/j.it.2019.05.003 31155315

[53] Keshari RS Jyoti A Dubey M Kothari N Kohli M Bogra J. Cytokines induced neutrophil extracellular traps formation: implication for the inflammatory disease condition. PloS One. (2012) 7:e48111. doi: 10.1371/journal.pone.0048111 23110185 PMC3482178

[54] Cristinziano L Modestino L Antonelli A Marone G Simon HU Varricchi G. Neutrophil extracellular traps in cancer. Semin Cancer Biol. (2022) 79:91–104. doi: 10.1016/j.semcancer.2021.07.011 34280576

[55] Korkmaz B Horwitz MS Jenne DE Gauthier F. Neutrophil elastase, proteinase 3, and cathepsin G as therapeutic targets in human diseases. Pharmacol Rev. (2010) 62:726–59. doi: 10.1124/pr.110.002733 PMC2993259 21079042

[56] Yoo DG Floyd M Winn M Moskowitz SM Rada B. NET formation induced by Pseudomonas aeruginosa cystic fibrosis isolates measured as release of myeloperoxidase-DNA and neutrophil elastase-DNA complexes. Immunol Lett. (2014) 160:186–94. doi: 10.1016/j.imlet.2014.03.003 24670966

[57] Zhu L Liu L Zhang Y Pu L Liu J Li X. High level of neutrophil extracellular traps correlates with poor prognosis of severe influenza A infection. J Infect Dis. (2018) 217:428–37. doi: 10.1093/infdis/jix475 29325098

[58] Modestino L Cristinziano L Poto R Ventrici A Trocchia M Ferrari SM. Neutrophil extracellular traps and neutrophil-related mediators in human thyroid cancer. Front Immunol. (2023) 14:1167404. doi: 10.3389/fimmu.2023.1167404 37705974 PMC10495767

[59] Stojanovic SK Stamenkovic BN Cvetkovic JM Zivkovic VG Apostolovic MRA. Matrix metalloproteinase-9 level in synovial fluid-association with joint destruction in early rheumatoid arthritis. Medicina (Kaunas). (2023) 59. doi: 10.3390/medicina59010167 PMC9863294 36676791

[60] Aratani Y. Myeloperoxidase: Its role for host defense, inflammation, and neutrophil function. Arch Biochem Biophys. (2018) 640:47–52. doi: 10.1016/j.abb.2018.01.004 29336940

[61] Fernandes RM da Silva NP Sato EI. Increased myeloperoxidase plasma levels in rheumatoid arthritis. Rheumatol Int. (2012) 32:1605–9. doi: 10.1007/s00296-011-1810-5 21331575

[62] Partsch G Steiner G BF L Dunky A Broll H Smolen JS. Highly increased levels of tumor necrosis factor-alpha and other proinflammatory cytokines in psoriatic arthritis synovial fluid. J Rheumatol. (1997) 24:518–23. 9058659

[63] Liu T Li S Ying S Tang S Ding Y Li Y. The IL-23/IL-17 pathway in inflammatory skin diseases: from bench to bedside. Front Immunol. (2020) 11:594735. doi: 10.3389/fimmu.2020.594735 33281823 PMC7705238

[64] Vecellio M Hake VX Davidson C Carena MC Wordsworth BP Selmi C. The IL-17/IL-23 axis and its genetic contribution to psoriatic arthritis. Front Immunol. (2020) 11:596086. doi: 10.3389/fimmu.2020.596086 33574815 PMC7871349

[65] Cross AL Hawkes J Frankland H Mediana A Wright HL Goodson NJ. Neutrophil function following treatment of psoriatic arthritis patients with secukinumab: altered cytokine signalling but no impairment of host defence. Rheumatol (Oxford). (2023) 62:3025–34. doi: 10.1093/rheumatology/kead007 36617171

[66] Siebert S Sweet KM Ritchlin CT Hsia EC Kollmeier AP Xu XL. Guselkumab modulates differentially expressed genes in blood of patients with psoriatic arthritis: results from two phase 3, randomized, placebo-controlled trials. ACR Open Rheumatol. (2023) 5:490–8. doi: 10.1002/acr2.11589 PMC10502816 37553909

[67] Keijsers RRMC Joosten I van Erp PEJ Koenen HJPM van de Kerkhof PCM. Cellular sources of IL-17 in psoriasis: a paradigm shift? Exp Dermatol. (2014) 23:799–803. doi: 10.1111/exd.12487 25039885

[68] Schon MP Erpenbeck L. The interleukin-23/interleukin-17 axis links adaptive and innate immunity in psoriasis. Front Immunol. (2018) 9:1323. doi: 10.3389/fimmu.2018.01323 29963046 PMC6013559

[69] Khmaladze I Kelkka T Guerard S Wing K Pizzolla A Saxena A. Mannan induces ROS-regulated, IL-17A-dependent psoriasis arthritis-like disease in mice. Proc Natl Acad Sci U.S.A. (2014) 111:E3669–78. doi: 10.1073/pnas.1405798111 PMC4156700 25136095

[70] Hayden H Ibrahim N Klopf J Zagrapan B Mauracher LM Hell L. ELISA detection of MPO-DNA complexes in human plasma is error-prone and yields limited information on neutrophil extracellular traps formed. vivo. PloS One. (2021) 16:e0250265. doi: 10.1371/journal.pone.0250265 33886636 PMC8062102

[71] Papayannopoulos V Zychlinsky A. NETs: a new strategy for using old weapons. Trends Immunol. (2009) 30:513–21. doi: 10.1016/j.it.2009.07.011 19699684

[72] Khandpur R Carmona-Rivera C Vivekanandan-Giri A Gizinski A Yalavarthi S Knight JS. NETs are a source of citrullinated autoantigens and stimulate inflammatory responses in rheumatoid arthritis. Sci Transl Med. (2013) 5:178ra40. doi: 10.1126/scitranslmed.3005580 PMC3727661 23536012

[73] Wigerblad G Kaplan MJ. Neutrophil extracellular traps in systemic autoimmune and autoinflammatory diseases. Nat Rev Immunol. (2023) 23:274–88. doi: 10.1038/s41577-022-00787-0 PMC9579530 36257987

[74] Ruiz-Limon P Ladehesa-Pineda ML Castro-Villegas MDC Abalos-Aguilera MDC Lopez-Medina C Lopez-Pedrera C. Enhanced NETosis generation in radiographic axial spondyloarthritis: utility as biomarker for disease activity and anti-TNF-alpha therapy effectiveness. J BioMed Sci. (2020) 27:54. doi: 10.1186/s12929-020-00634-1 32303225 PMC7164280

[75] Perez-Sanchez C Ruiz-Limon P Aguirre MA Jimenez-Gomez Y Arias-de la Rosa I Abalos-Aguilera MC. Diagnostic potential of NETosis-derived products for disease activity, atherosclerosis and therapeutic effectiveness in Rheumatoid Arthritis patients. J Autoimmun. (2017) 82:31–40. doi: 10.1016/j.jaut.2017.04.007 28465139

[76] Divangahi M Aaby P Khader SA Barreiro LB Bekkering S Chavakis T. Trained immunity, tolerance, priming and differentiation: distinct immunological processes. Nat Immunol. (2021) 22:2–6. doi: 10.1038/s41590-020-00845-6 33293712 PMC8020292

