# Supplementary material for: Neutrophil exhaustion and impaired functionality in psoriatic arthritis patients
Source: Front Immunol. 2024 Sep 6;15:1448560. doi: 10.3389/fimmu.2024.1448560 (PMC11412820; doi:10.3389/fimmu.2024.1448560)
Supplement: Supplementary file 1 [file DataSheet1.docx]

**
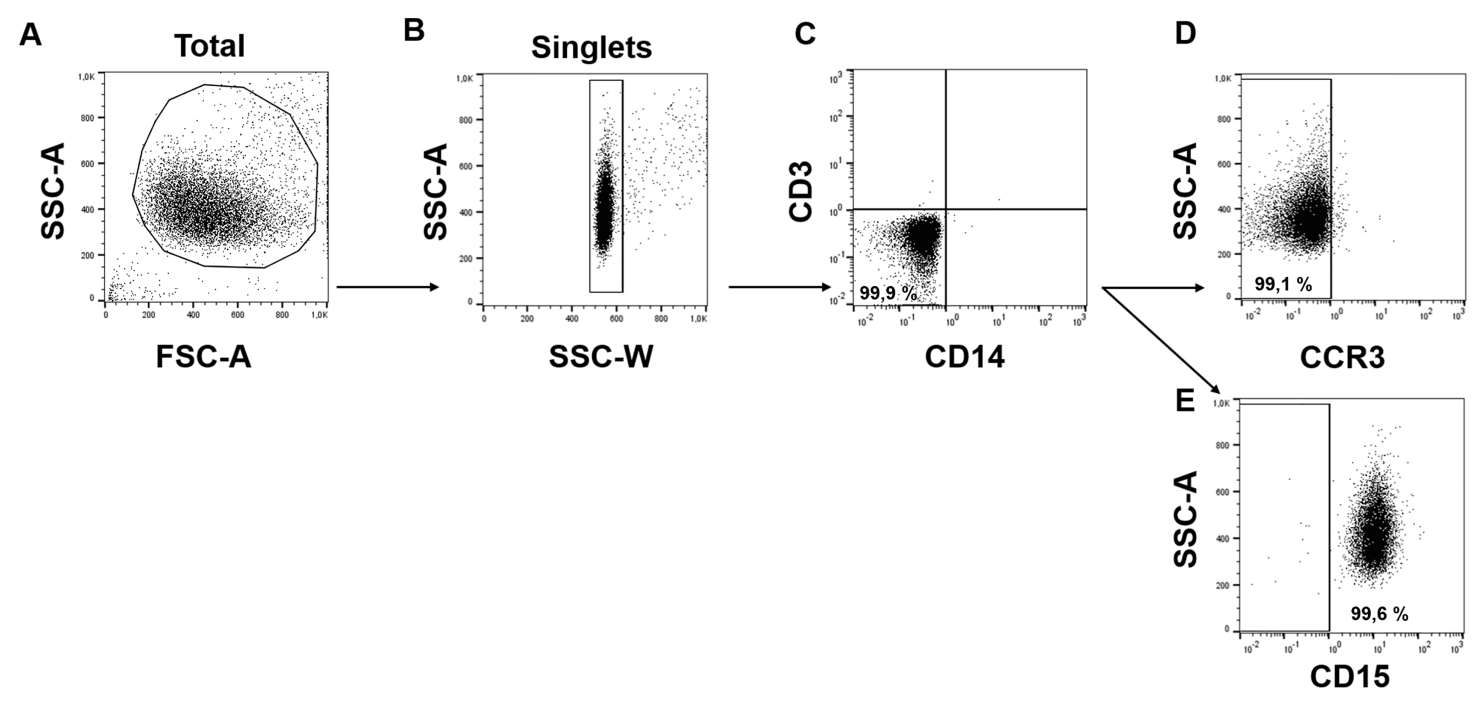
**

**Supplementary Figure 1.** PMNs were purified from the peripheral blood of HCs and/or PsA patients and stained with the monoclonal antibodies as indicated. Flow cytometry plots were gated on live cells and show forward (FSC) and side scatter (SSC) of EasySep-purified untouched neutrophils (**A, B**). PMNs were negative for CD3, CD14, and CCR3 (**C, D**), and positive for CD15 (**E**).

**
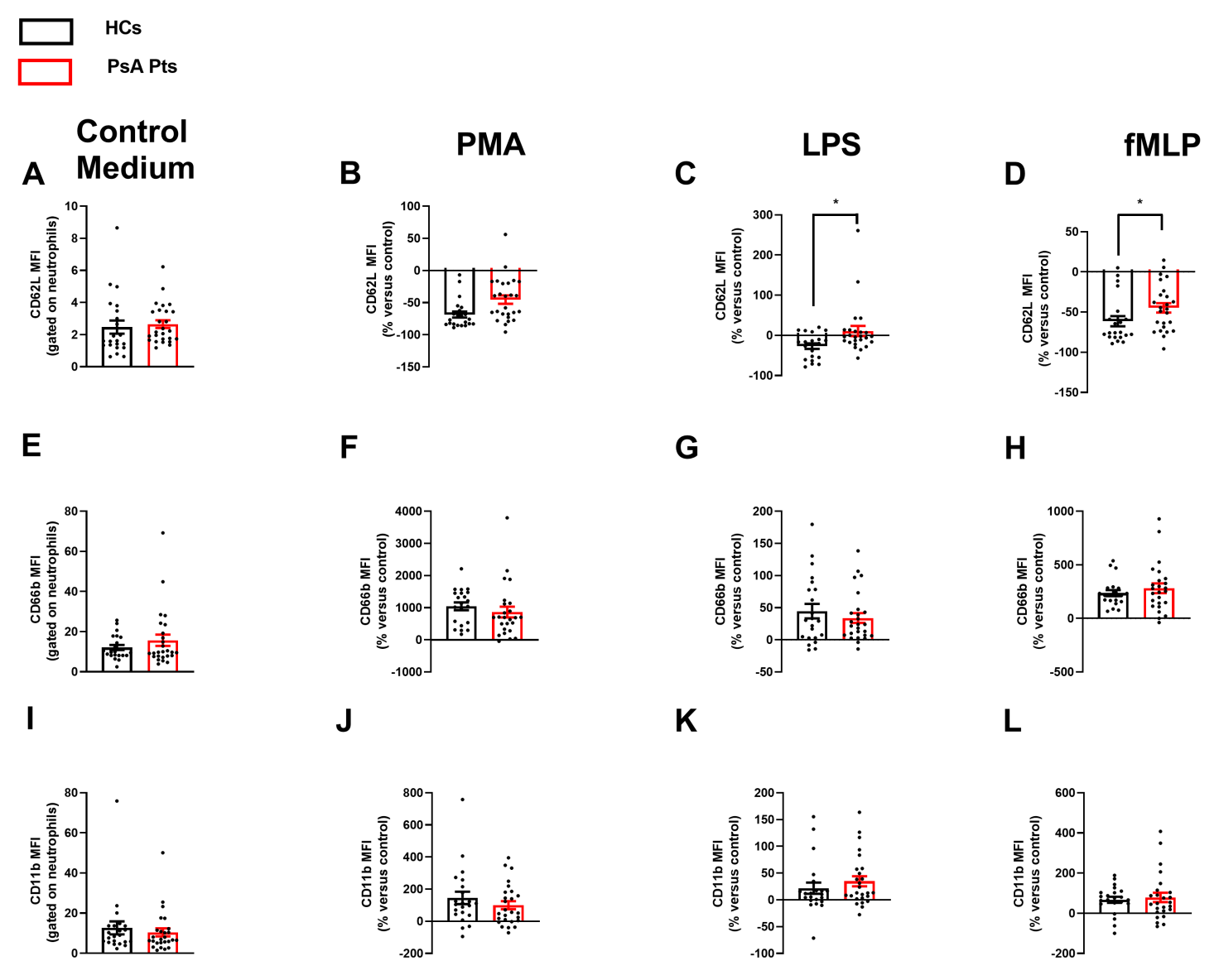
**

**Supplementary Figure 2.** PMNs from peripheral blood of PsA patients (PsA Pts, red borders) and HCs (HCs, black borders) were stimulated with control medium (**A, E, I**), PMA (10 ng/mL) (**B, F, J**), LPS (100 ng/mL) (**C, G, K**), and fMLP (1 µM) (**D, H, L**) for 60 min at +37 °C, then stained for the neutrophil activation markers CD62L (**A–D**), CD66b (**E–H**), and CD11b (**I–L**) and subjected to cytofluorimetric analysis. Each point in graphs A–L represents one patient (PsA Pts) or one healthy control (HCs). Mean fluorescence intensity (MFI) of CD62L, CD66b, and CD11b was calculated and expressed as raw data for control medium or normalized for non-stimulated cells (control medium) for PMA, LPS, or fMLP stimulation (mean ± SEM); **p* < 0.05, analyzed by Student’s-t-test or Mann-Whitney U test, depending on the distribution of the data.

**
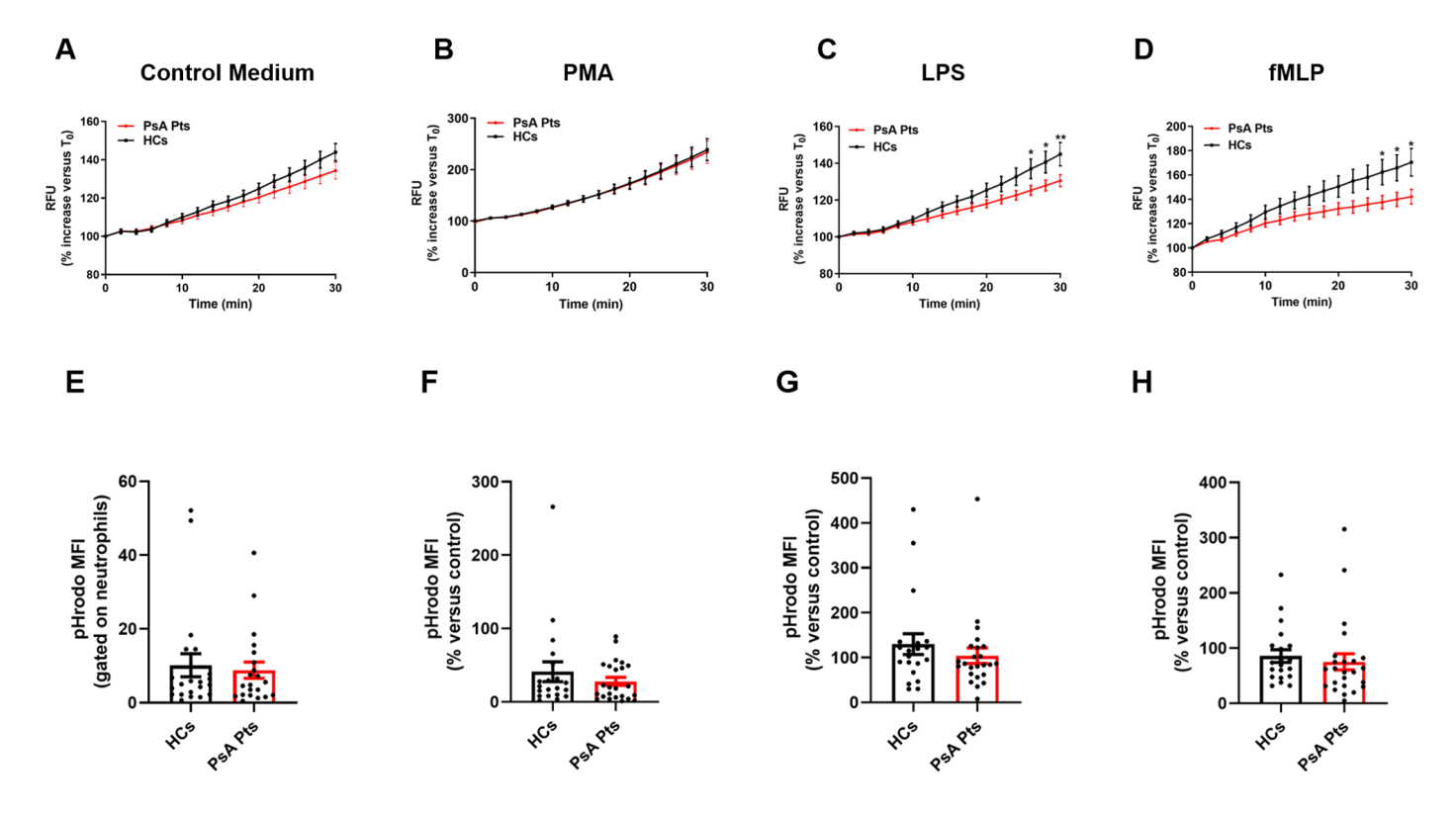
**

**Supplementary Figure 3.** PMNs from peripheral blood of PsA patients (red lines) and HCs (black lines) were incubated (30 min, +37 °C) with 2’,7’-dichlorodihydrofluorescein diacetate (H_2_DCFDA, 10 µM), washed and then stimulated with control medium (**A**), PMA (10 ng/mL) (**B**), LPS (100 ng/mL) (**C**) and fMLP (1µM) (**D**). Immediately after stimulation, PMNs were analyzed with a multimode microplate reader (EnSpire Multimode Plate reader, PerkinElmer), and DCF fluorescence was measured for 30 min at 2 min intervals. The results were expressed as RFU and percentage increase *versus* time 0 (mean ± SEM); **p* < 0.05; ***p* < 0.01, analyzed by two-way ANOVA and Bonferroni post-test. **E–H.** PMNs from peripheral blood of PsA patients (PsA Pts, red border) and HCs (HCs, black border) were stimulated with control medium (**E**), PMA (10 ng/mL) (**F**), LPS (100 ng/mL) (**G**), or fMLP (1 µM) (**H**) for 60 min at +37 °C and then incubated with pHrodo^TM^ Green *E. coli* BioParticles® conjugate (100 µg/mL) for 60 min at +37 °C, with or without cytochalasin B (as inhibitor of phagocytosis, 10 µM), followed by flow cytometry analysis. MFI of cells positive for pHrodo^TM^ was calculated and expressed as raw data for control medium or normalized for non-stimulated cells (control medium) for PMA, LPS, or fMLP stimulation (mean ± SEM), analyzed by Student’s-t-test or Mann-Whitney U test, depending on the distribution of the data.

**
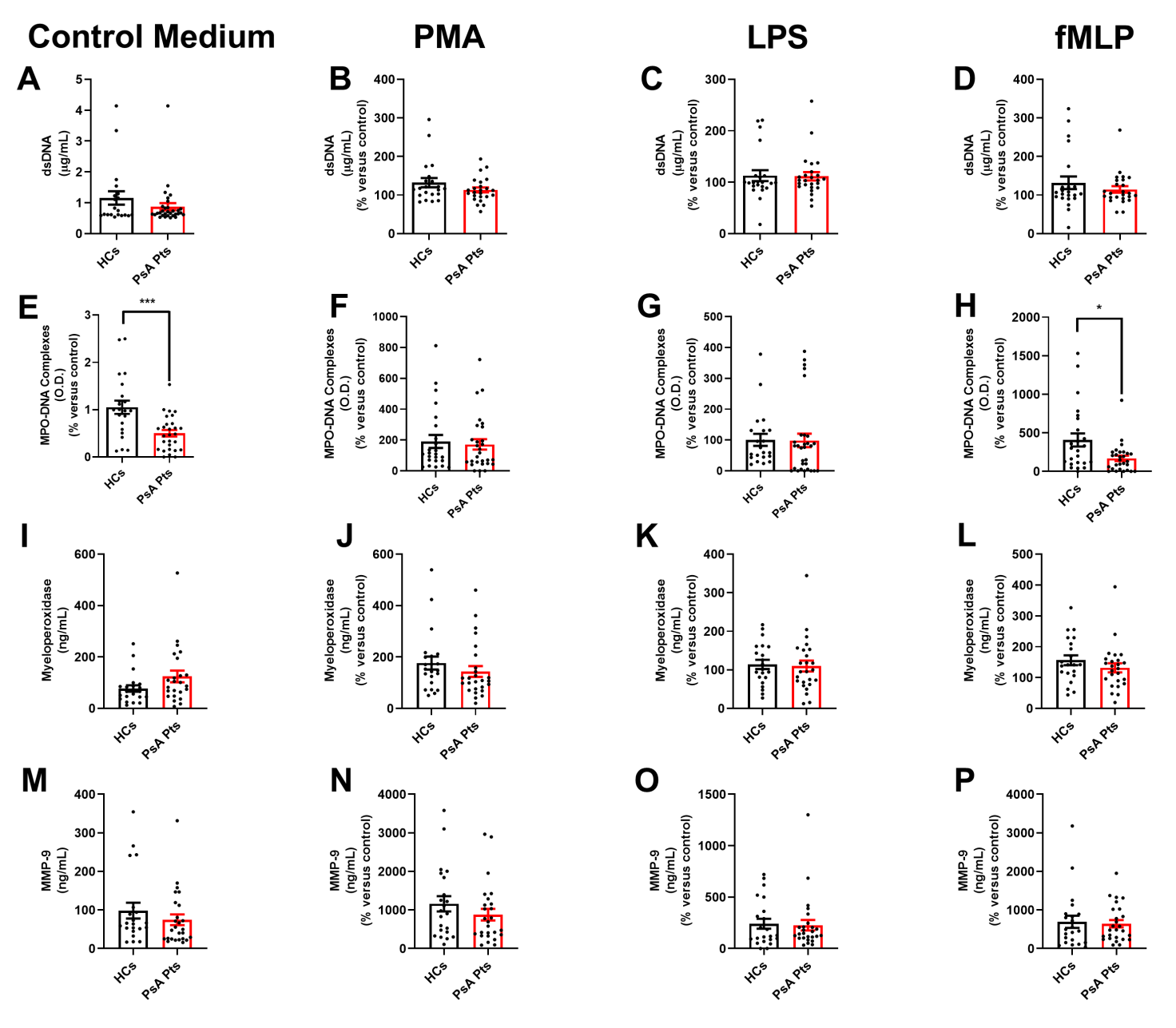
**

**Supplementary Figure 4.** PMNs from peripheral blood of PsA patients (PsA Pts, red borders) and HCs (HCs, black borders) were cultured for 60 min at 37 °C in the presence of control medium (**A, E, I, M**), PMA (10 ng/mL) (**B, F, J, N**), LPS (100 ng/mL) (**C, G, K, O**), and fMLP (1 µM) (**D, H, L, P**). The extracellular levels of dsDNA (**A–D**), MPO-DNA complexes (**E–H**), MPO (**I–L**), and MMP-9 (**M–P**) were measured by Quant-iT^TM^ PicoGreen^TM^ dsDNA Assay Kit or by ELISA, respectively. The extracellular levels of dsDNA, MPO-DNA complexes, MPO, and MMP-9 were calculated. Results were expressed as raw data for control medium or normalized for non-stimulated cells (control medium) for PMA, LPS, or fMLP stimulation (mean ± SEM). **p* < 0.05; *** *p* < 0.005, analyzed by Student’s-t-test or Mann-Whitney U test, depending on the distribution of the data.

**
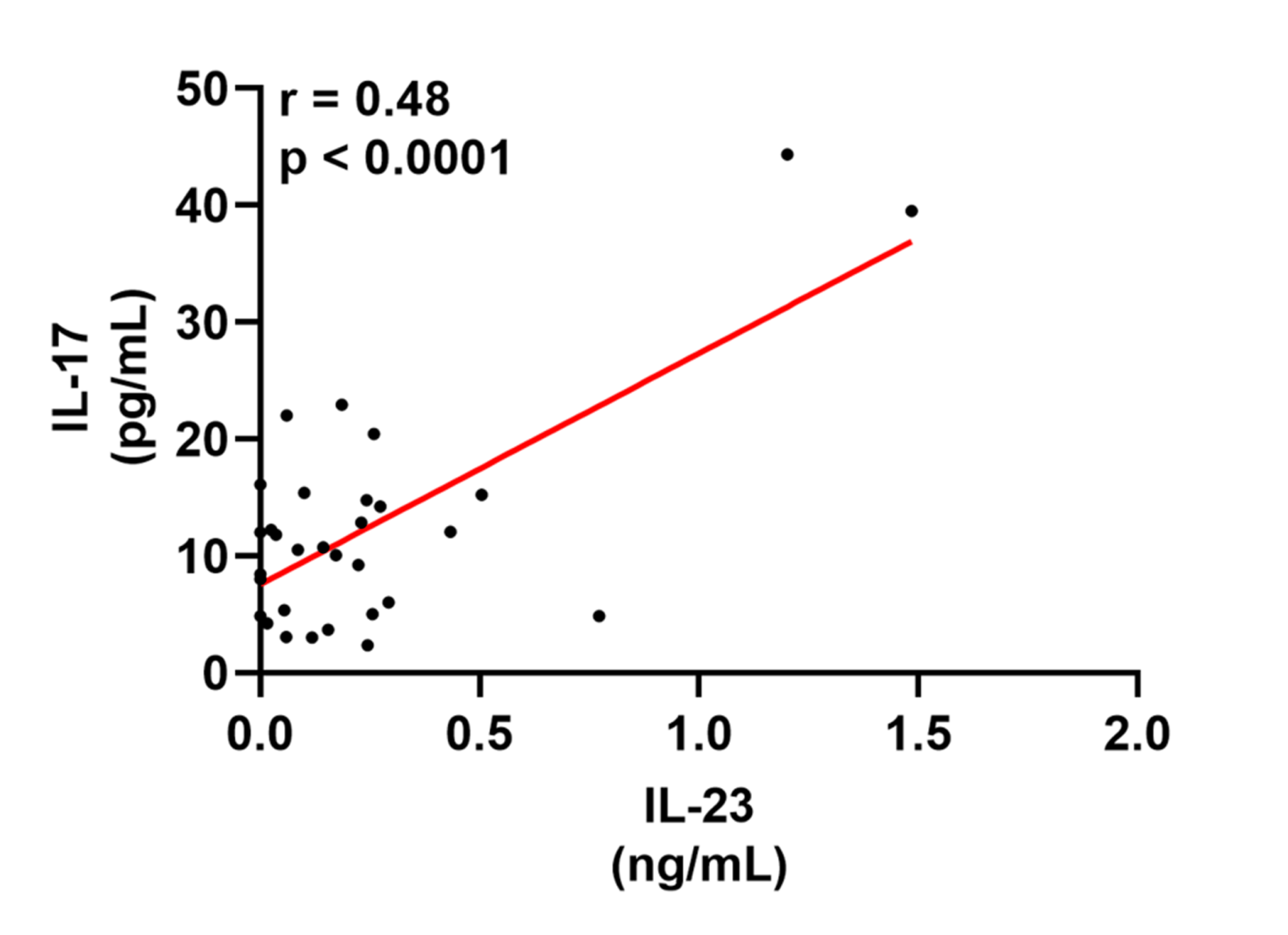
**

**Supplementary Figure 5.** Correlation between serum concentrations of IL-17 and IL-23 in PsA patients. Spearman correlation test; *r* = 0.48; *p* < 0.0001.
